# Kawasaki disease following a pediatric scald burn: a case report and literature review

**DOI:** 10.3389/fped.2026.1902593

**Published:** 2026-08-19

**Authors:** Hanqin Ye, Kun Yang, Binjie Luo, Geng Ji, Xiaopeng Zheng

**Affiliations:** Department of Burn Surgery, Taizhou School of Clinical Medicine, Nanjing Medical University, Taizhou, Jiangsu, China

**Keywords:** case report, coronary artery lesion, Kawasaki disease, mucocutaneous lymph node syndrome, pediatric burns

## Abstract

**Background:**

Kawasaki disease (KD) is a systemic medium-vessel vasculitis of early childhood that, rarely, has been reported during the course of a pediatric burn. Because persistent fever, rash, and mucocutaneous changes overlap with the systemic inflammatory response to a burn wound, KD can go unrecognized, delaying the administration of intravenous immunoglobulin (IVIG), which reduces the risk of coronary sequelae.

**Case description:**

A previously healthy 1-year-old boy sustained a 6% total body surface area deep partial-thickness scald to the left lower limb. The wound healed with conservative dressing care and was almost completely re-epithelialized (>99%) by postburn day 10. On that same day, he developed an abrupt, persistent fever that peaked at 39.5°C–40°C and did not respond to empirical antibiotics. Over the next 48 h, he developed bilateral non-purulent bulbar conjunctival injection, erythematous cracked lips with a strawberry tongue, a polymorphous trunk rash, palmoplantar erythema with hand induration, and non-suppurative cervical lymphadenopathy (largest node 20 × 15 mm). Investigations showed neutrophilic leukocytosis (WBC 16.22 × 10⁹/L), a C-Reactive Protein (CRP) level of 42.65 mg/L, mild hyperbilirubinemia and transaminitis, and elevated levels of IL-6, soluble IL-2 receptor, and TNF-α. The platelet count was 456 × 10⁹/L at admission, 384 × 10⁹/L during the acute phase, and increased to 657 × 10⁹/L during convalescence. Infectious and rheumatological differentials were excluded. The patient met all six principal criteria of the JCS/JSCS 2020 guideline, leading to a diagnosis of complete KD. He received IVIG (2 g/kg) and oral aspirin (30 mg/kg/day, tapered after defervescence). Fever resolved within 24 h, followed by periungual desquamation. Transthoracic echocardiography showed a patent foramen ovale without coronary artery abnormalities, and repeat imaging at approximately 1 week and 1 month after onset confirmed normal coronary arteries with no evidence of aneurysms.

**Conclusion:**

This case illustrates that KD can emerge as a burn wound re-epithelializes, at which point the reappearance of fever is easily misattributed to an intercurrent infection. Across the present case and 13 previously reported, primary-source-verified cases, burn-associated KD occurred in children aged 5 years or younger, usually presented within about 2 weeks of the burn, mostly followed modest burns, and responded promptly to IVIG, with acute coronary artery changes observed in a minority (three of 14) that regressed after treatment. Whether burn injury triggers KD or the two co-occur by chance cannot be determined from a single case; the practical message is that clinicians should re-examine all mucocutaneous surfaces and consider KD in any pediatric burn patient with unexplained persistent fever, so that IVIG is not delayed.

## 1 Introduction

Kawasaki disease (KD), or mucocutaneous lymph node syndrome, is an idiopathic, usually self-limiting systemic vasculitis of medium-sized arteries that predominantly affects children under 5 years of age (1). Its coronary artery lesions are the leading cause of acquired heart disease in children in developed countries, and timely intravenous immunoglobulin (IVIG) markedly reduces that risk; however, the early presentation is non-specific and recognition is often delayed (2–5).

KD arising during a pediatric burn is rare, with only a small number of cases reported worldwide (6–12). In this setting, the cardinal features of KD (persistent fever, rash, and mucocutaneous changes) may be masked by the burn's own inflammatory response, wound care, or analgesia, leading to easy misdiagnosis and exposing the child to avoidable cardiovascular risk.

We report a 1-year-old boy who developed complete KD as a deep partial-thickness scald re-epithelialized, and we place the case alongside previously published cases of burn-associated KD. Our aim is descriptive: to identify the shared clinical features that may prompt earlier recognition, rather than to assert a causal link. Reports were identified through PubMed, Science Direct, CiNii, and J-STAGE using terms combining “Kawasaki disease” with “burn,” “scald,” or “thermal injury” (searched through May 2025). After merging duplicate reports of the same patients and excluding four Japanese-language cases whose primary sources could not be retrieved, 13 primary-source-verified cases remained for comparison (Table 1). The report follows the CARE guideline (13).

**Table 1:** Comparison of the present case with 13 previously reported, primary-source-verified cases of burn-associated Kawasaki disease.

Case	First author (year), country	Age/sex	TBSA %/depth	Fever onset (postburn day)	KD criteria (/6)	Peak WBC (×10⁹/L)/CRP (mg/L)	Wound culture	Coronary artery findings (acute)	Defervescence after IVIG (days)
1^a^	Present case (2026), China	1 year/M	6/II	10	6/6	16.22/42.65	Not recultured at fever onset	PFO; no CAL (normal at 1 month)	1
2	Wong (2004) (6), Canada	2 years/M	9/I–II	8	6/6	↑/↑	Negative	Normal	7
3	Wong (2004) (6), Canada	2 years/M	20/II	2	5/6	Normal/↑	Negative	LMCA dilatation	7
4	Ito (2006) (7), Japan	5 y/M	2/II	2	5/6	19.80/39.2	Negative	Normal	7
5	Muro (2009) (8), Japan	11 months/M	20/II	7	5/6	10.60/17.3	*E. coli*	Normal	5
6	Muro (2009) (8), Japan	23 months/M	10/II	11	5/6	14.70/10.8	Negative	Normal	5
7	Muro (2009) (8), Japan	2 years/M	1/II	3	6/6	7.30/8.7	Negative	Normal	NR
8	Muro (2009) (8), Japan	13 months/F	20/I–II	7	5/6	17.6/12.6	Negative	Normal	5
9	Feng (2011) (9), China	17 months/F	61/I–II	4	6/6	14.0/15.3	Negative	LMCA mild dilatation	7
10	Feng (2011) (9), China	10 months/M	20/I–II	4	4/6	24.5/10.1	MSSA/MRSA	CAL	NR
11	Feng (2011) (9), China	2 years 2 months/F	0.5/II	19	5/6	NR	MRSA	Normal	NR
12^b^	Okada (2020) (10), Japan	17 months/F	sunburn/I–II	5	6/6	NR/↑	—	Normal	1
13	Shimabukuro (2022) (11), Japan	11 months/M	10.5/II	2	6/6	NR	MRSA (non-TSST-1)	Normal	1
14	Ueda (2018) (12), Japan	8 months/F	13/II	5	5/6	NR	Negative	Normal	2

a Present case.

b Non-thermal UV injury (severe sunburn); mechanistically distinct. It is retained in the descriptive pooling for completeness, and the observed patterns remain unchanged if it is excluded. Blood cultures were negative in all cases where obtained. All patients defervesced after IVIG ± aspirin, and where follow-up coronary imaging was reported, the coronary changes regressed.

CAL, coronary artery lesion; LMCA, left main coronary artery; PFO, patent foramen ovale; NR, not reported; ↑, reported as elevated without a numeric value. Four additional Japanese-language cases (two reported by Kojima 2000 and two reported by Nakagawa 2010), summarized in Ueda et al., were excluded because the primary sources were unretrievable.

## 2 Case description

### 2.1 Admission

A previously healthy 1-year-old boy (9 kg) presented 4 h after a hot-water scald to the left lower limb. There was no relevant personal or family history and no recent infection, vaccination, or travel. On admission, he was alert but distressed, with a heart rate of 130/min, a respiratory rate of 26/min, and an axillary temperature of 37.5°C. The burn covered approximately 6% total body surface area (TBSA) (estimated using the Lund–Browder chart) over the left calf and dorsum of the foot, with a mixed red-and-white blistered wound bed and no surrounding erythema or induration. Admission laboratory results are summarized in Table 2; urinalysis, stool examination, and coagulation were normal, and viral hepatitis serology was negative except for a positive hepatitis B surface antibody. Electrocardiography showed sinus arrhythmia. An admission diagnosis of deep partial-thickness scald, 6% TBSA, to the left lower limb was made.

**Table 2:** Laboratory results at admission, on day 10, and on day 20 after injury.

Parameter (unit)	At admission	On day 10	On day 20	Laboratory reference intervals
Red blood cells (×10^12^/L)	4.00	3.77	4.02	4.3–5.8
Hematocrit (%)	31.2	29.7	33.1	40–50
Hemoglobin (g/L)	108	100	106	130–175
White blood cells (×10⁹/L)	9.25	16.22	8.08	3.5–9.5
Absolute neutrophils (×10⁹/L)	4.02	11.97	2.03	1.8–6.3
Absolute lymphocytes (×10⁹/L)	4.16	3.36	4.40	1.1–3.2
Absolute monocytes (×10⁹/L)	0.70	0.52	1.27	0.1–0.6
Platelets (×10⁹/L)	456	384	657	125–350
C-reactive protein (mg/L)	<0.499	42.65	<0.499	0–10
Total bilirubin (μmol/L)	12.0	23.7	8.1	0–23
Direct bilirubin (μmol/L)	3.1	7.2	2.8	0–6.8
Indirect bilirubin (μmol/L)	8.9	16.5	5.3	0–19
Albumin (g/L)	39.9	37.9	39.6	40–55
Aspartate aminotransferase (U/L)	50	51	819	15–40
Alanine aminotransferase (U/L)	20	39	699	9–50

### 2.2 Clinical course and emergence of KD

**Figure 1:**
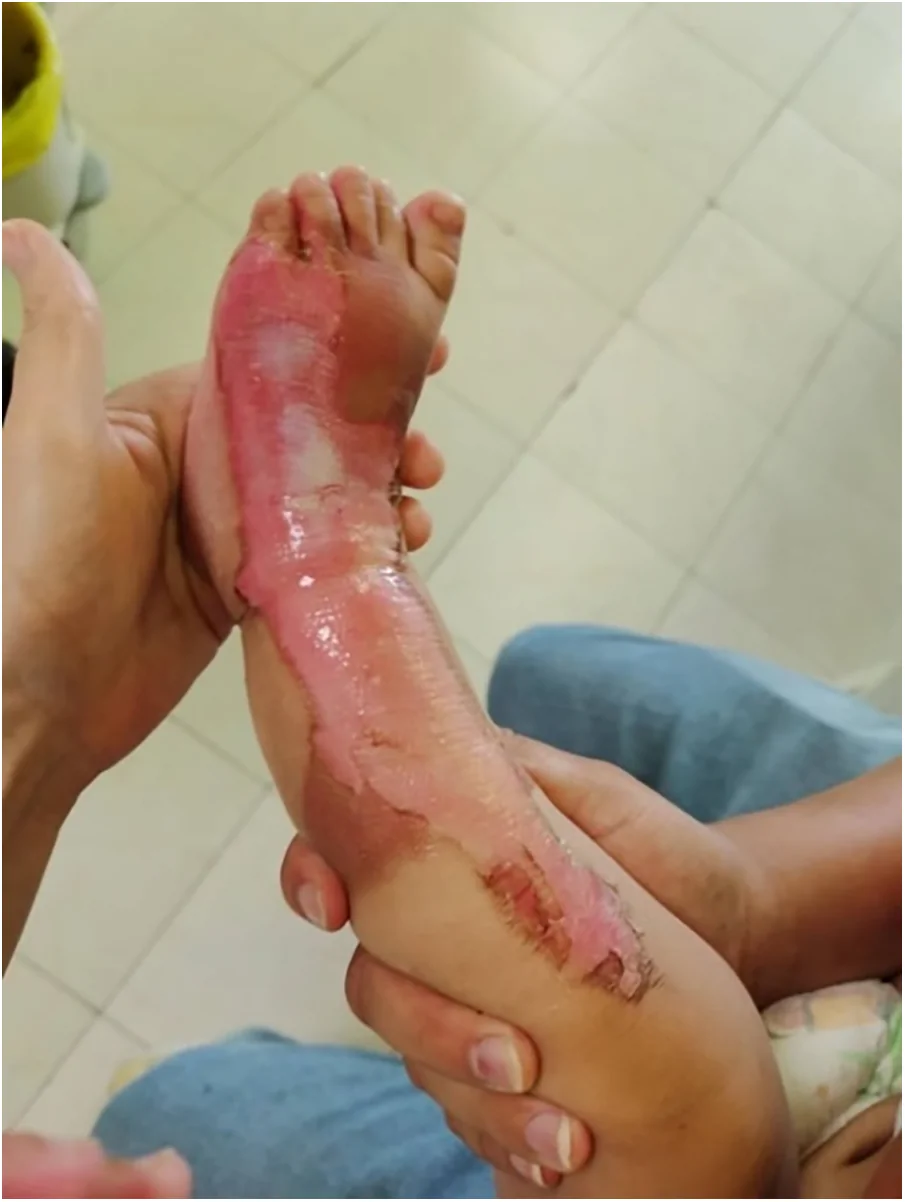
Burn wound on postinjury day 10. The 6% TBSA scald on the left lower limb shows near-complete re-epithelialization (>99% of wound surface); only minimal residual necrotic tissue is attached, and no clinical signs of local infection are evident.

Dressings were changed every 2–3 days; the child stayed afebrile and stable. By postburn day 10, the wound was >99% re-epithelialized with a small area of clean granulation and no sign of local infection (Figure 1).

On that day, he abruptly developed a fever that peaked at 39.5°C, with listlessness, an occasional cough, rhinorrhoea, and poor appetite; examination showed only oropharyngeal injection. Repeat testing showed neutrophilic leukocytosis, a CRP level of 42.65 mg/L, and mild elevations in bilirubin and Aspartate Aminotransferase (AST) (Table 2). Empirical intravenous cefotaxime was started for a presumed upper respiratory infection, with oral antipyretics.

Fever persisted despite 48 h of antibiotics, peaking at 40°C. On postburn day 12, a full mucocutaneous examination disclosed the complete picture: bilateral non-purulent bulbar conjunctival injection with watery discharge; erythematous, dry, cracked lips with a strawberry tongue (prominent fungiform papillae); oropharyngeal injection; a scattered blanching erythematous maculopapular rash on the trunk; palmoplantar erythema with mild induration of both hands; and bilateral non-suppurative cervical lymphadenopathy (largest node 20 mm × 15 mm). There was no extremity edema, no perianal desquamation, and the Bacillus Calmette-Guérin (BCG) inoculation site was unremarkable. Because the scalded limb was under dressings, the palmoplantar and extremity changes were only appreciated when all four limbs were deliberately unwrapped and examined.

Extended testing (Table 3) showed elevated IL-6 (110 pg/mL), soluble IL-2 receptor (2,158 U/mL), TNF-α (23.1 pg/mL), and IL-10 (12.6 pg/mL); a CD4⁺/CD8⁺ ratio of 3.70; and elevated fibrinogen (6.87 g/L) and D-dimer (1,340 ng/mL). Blood cultures, antistreptolysin O, humoral immunity panels, Epstein–Barr virus serology, ferritin, and heparin-binding protein were unremarkable. Transthoracic echocardiography showed a patent foramen ovale (an incidental congenital finding unrelated to KD) without coronary artery dilatation, with coronary internal diameters remaining within normal limits for body surface area. Abdominal ultrasound showed mild hepatomegaly. The platelet count, which was normal at admission (456 × 10⁹/L) and during the acute febrile phase (384 × 10⁹/L), later rose to 657 × 10⁹/L in convalescence (Table 2), consistent with the thrombocytosis characteristic of the second week of KD.

**Table 3:** Inflammatory cytokines, lymphocyte subsets, and fibrinolytic markers on postinjury day 10.

Category	Parameter (unit)	Result	Laboratory reference intervals
Inflammatory cytokines	Serum IL-1 (pg/mL)	<5.00	<20
	Soluble IL-2 receptor (U/mL)	2,158	398–1940
	Serum IL-6 (pg/mL)	110	0–3.26
	TNF-α (pg/mL)	23.1	3.96–12.30
	Serum IL-10 (pg/mL)	12.6	1.82–7.19
Lymphocyte subsets	Helper/inducer T cells (%)	45.03	30–55
	Suppressor/cytotoxic T cells (%)	12.16	15–35
	NK cells (%)	4.46	5–25
	B lymphocytes (%)	27.07	10–35
	CD4⁺/CD8⁺ ratio	3.70	1.2–6.2
	Total T cell count (cells/μL)	2,100.22	998–3,975
	Helper/inducer T cells (cells/μL)	1,442.51	420–1,974
	NK cell count (cells/μL)	153.79	53–316
	B cell count (cells/μL)	933.95	272–1,510
Fibrinolytic system	Fibrinogen (g/L)	6.87	1.5–4.0
	D-dimer (mg/L)	1.34	≤1.2
	Fibrinogen Degradation Products (FDP) (μg/mL)	15.77	0–5

### 2.3 Diagnosis and treatment

Measles, scarlet fever, Epstein–Barr virus infection, streptococcal pneumonia, multisystem inflammatory syndrome in children (MIS-C), systemic lupus erythematosus, systemic juvenile idiopathic arthritis, and systemic bacterial infection were excluded on clinical and laboratory grounds. MIS-C was considered unlikely: the child had no gastrointestinal-predominant illness, hemodynamic instability, or ventricular dysfunction, and fulfilled the classic KD criteria (with a negative SARS-CoV-2 PCR/serology result or no known recent SARS-CoV-2 exposure). The patient met all six principal criteria of the JCS/JSCS 2020 guideline (14): prolonged fever, bilateral bulbar conjunctival injection, lip and oral changes, polymorphous exanthem, extremity changes, and non-suppurative cervical lymphadenopathy. Complete KD was therefore diagnosed.

**Figure 2:**
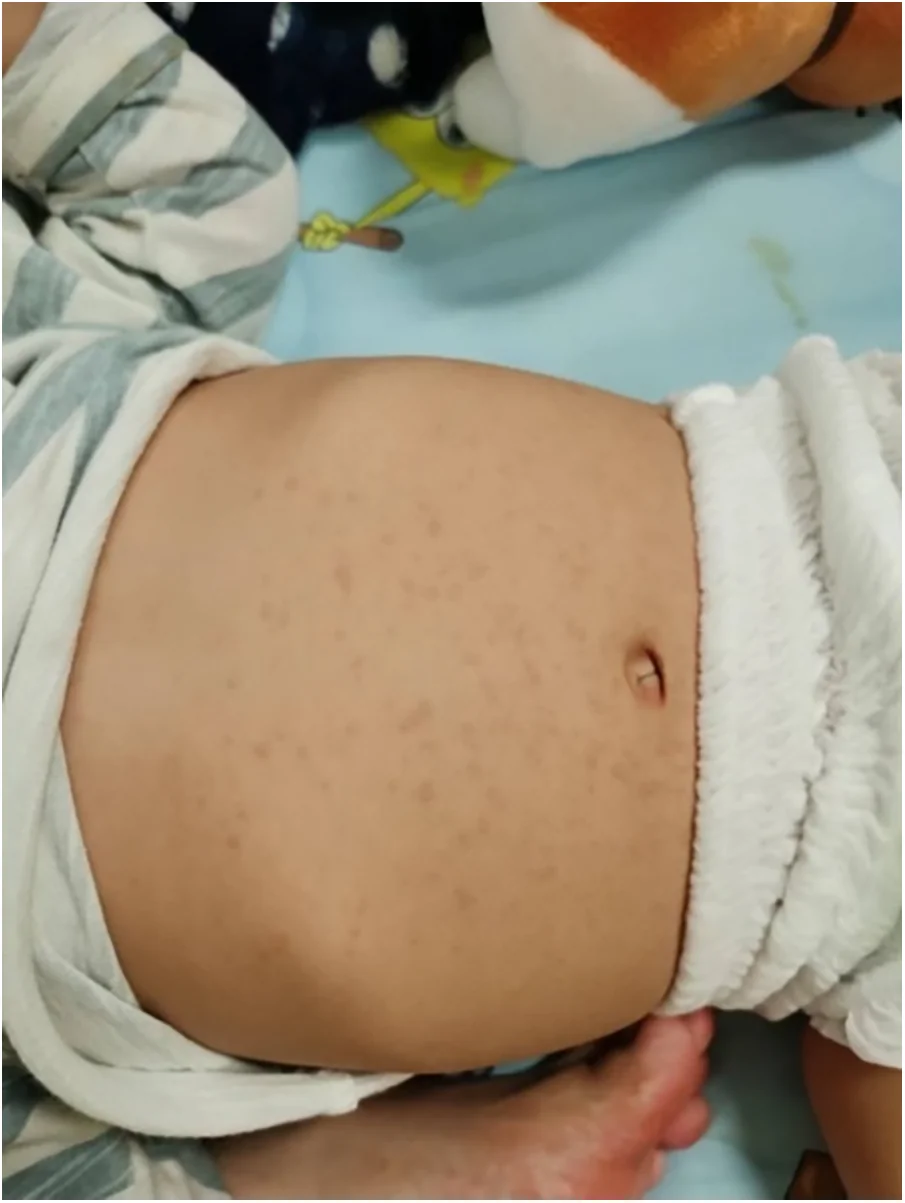
Trunk rash in the patient with Kawasaki disease. Polymorphous blanching erythematous maculopapular rash, shown to gradually subside after intravenous immunoglobulin and aspirin therapy.

**Figure 3:**
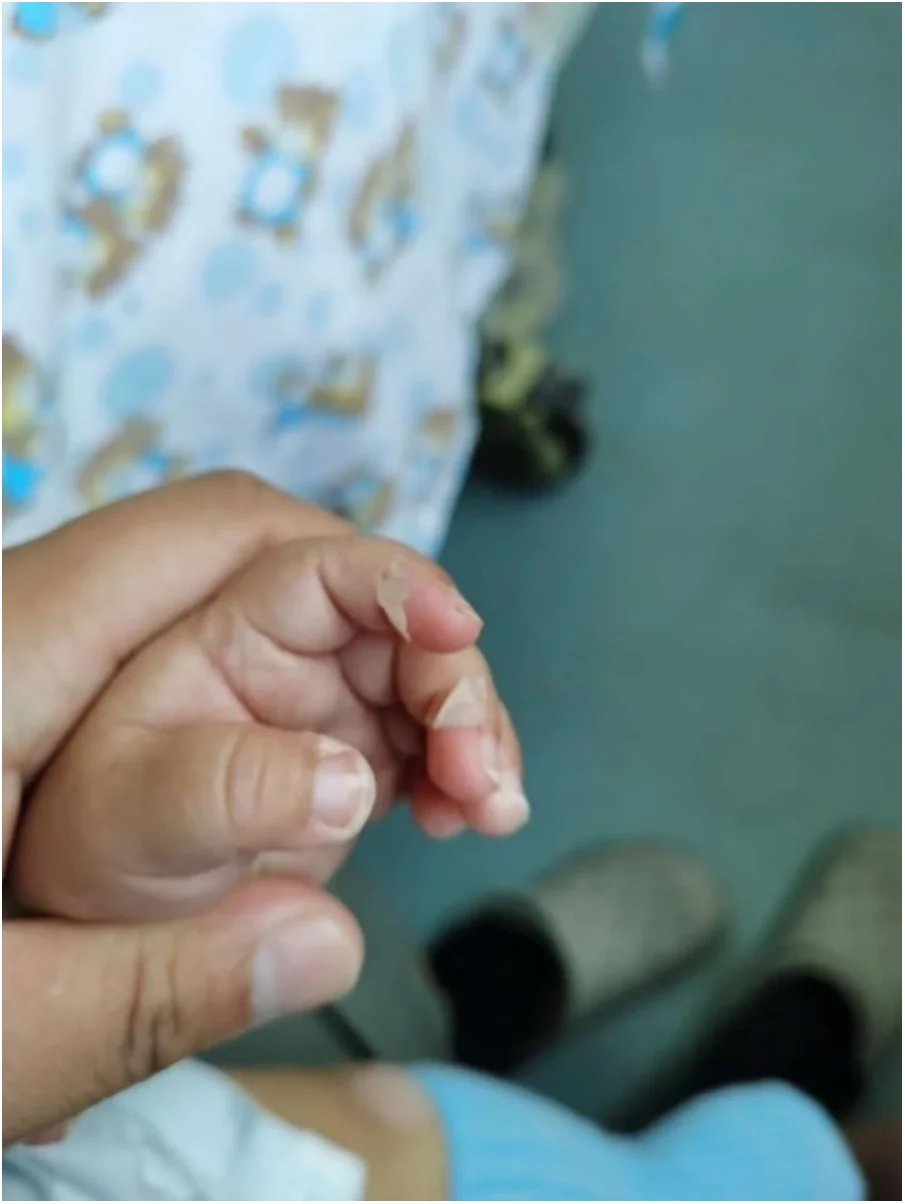
Membranous periungual desquamation of the fingertips during the convalescent phase of Kawasaki disease.

On postburn day 12, which was the third day of fever, he received a single IVIG infusion (2 g/kg over 12 h) and oral aspirin (30 mg/kg/day in four divided doses), alongside dipyridamole, oral fructose-1,6-diphosphate for myocardial support, and supportive care; burn dressings were continued every 2 days. Fever settled within 24 h of the IVIG infusion, and aspirin was reduced to 3–5 mg/kg/day after 48 h of being afebrile. The rash resolved (Figure 2), and membranous periungual desquamation appeared during convalescence (Figure 3).

During admission, repeat echocardiography approximately 1 week after disease onset again showed normal coronary arteries. He was discharged 30 days after injury with full wound healing and resolution of all signs of KD, continuing low-dose aspirin, dipyridamole, and fructose-1,6-diphosphate. About 2 weeks after discharge (approximately 1 month after disease onset), complete blood counts, CRP levels, Erythrocyte Sedimentation Rates (ESRs), biochemistry profiles, chest radiographs, ECGs, and echocardiograms were all normal, revealing normal coronary arteries and no evidence of aneurysms (day 20 laboratory values are presented in Table 2).

### 2.4 Patient (parent) perspective

The parents recalled being especially anxious when high fever returned just as the burn wound had nearly healed, having assumed the child was recovering. They expressed relief and gratitude that Kawasaki disease was recognized and treated before any heart complications developed.

## 3 Discussion

The instructive feature of this case is *timing*. KD began exactly as the burn wound re-epithelialized, once local infection had settled and inflammatory markers had normalized. The abrupt return of fever was therefore attributed, understandably, to an intercurrent upper respiratory infection. Only when the fever persisted for 48 h despite antibiotics and a deliberate, whole-body mucocutaneous re-examination was performed did the complete KD picture become apparent. A second practical lesson follows from the first: the dressed limb concealed the palmoplantar and extremity signs, which were recognized only when all limbs were unwrapped and examined. In a febrile burned child, the affected part is the one most likely to conceal diagnostic signs, and it is the part least often re-examined.

Set against the 13 previously reported, primary-source-verified cases (Table 1), this patient fits the established pattern while adding detail at its edges. *Age*: all 14 patients were 5 years of age or younger, most between 8 months and 2 years, the window in which passive maternal immunity wanes. *Timing of fever*: onset clustered between postburn days 2 and 11, overlapping the edema-reabsorption and acute phases; our patient (day 10) sits at the later end, and a single literature outlier presented on day 19 (9). *Burn size*: most were modest (≤20% TBSA), the sole severe burn being a 61% TBSA infant (9); our 6% scald is representative. *Microbiology*: wound cultures were often negative, and where organisms grew, they were inconsistent (*Escherichia coli*; MSSA/MRSA; one MRSA confirmed non-toxin-producing), so no single pathogen explains the association. *Coronary involvement and outcome*: transient coronary changes were documented acutely in three of 14 (cases 3, 9, and 10), all of which responded to IVIG, while our patient had no coronary lesions. What our case contributes beyond prior reports is a broad, objective immunological profile (markedly elevated soluble IL-2 receptor, IL-6, and TNF-α, with a shifted CD4⁺/CD8⁺ ratio) documented at presentation. These markers are non-specific and cannot by themselves confirm a diagnosis of exclusion, but they are consistent with the systemic immune activation of KD and document its intensity.

Why KD should occasionally accompany a burn remains unknown, and this single case cannot resolve the question. Current models of KD invoke an environmental, most likely infectious, trigger acting on a genetically susceptible child (15, 16). Three burn-specific hypotheses have been proposed but remain unproven: that loss of the cutaneous barrier permits bacterial superantigens (e.g., staphylococcal TSST-1, streptococcal pyrogenic exotoxins) to reach the circulation and drive polyclonal T-cell activation (17, 18); that absorption of damage-associated molecular patterns from devitalized tissue provides a sterile immune trigger [HMGB1 was elevated in the sunburn-associated case of Okada et al. (10)]; and that postburn immune dysregulation, with monocyte NF-*κ*B activation and IL-6/TNF-α/MMP-9 release, injures the vascular endothelium (19–22). The cytokine profile of our patient is consistent with such activation, but no superantigen or Damage-Associated Molecular Pattern (DAMP) testing was performed, and the trigger remains unknown. In the present case, these wound-based mechanisms in fact fit poorly: the burn was small (6% TBSA), uninfected, and almost fully healed when KD began, which further favors coincidence over a burn-driven trigger. Importantly, the short latency between burn and KD can equally be read as coincidental co-occurrence rather than causation; we therefore make no causal claim.

One laboratory observation deserves a brief, practical note: transaminases were near-normal on day 10 (AST 51, Alanine Aminotransferase (ALT) 39 U/L) but rose sharply by day 20 (AST 819, ALT 699 U/L) before normalizing by discharge. Likely contributors are high-dose aspirin hepatotoxicity and transient KD-associated hepatic involvement; notably, the transaminase peak occurred after the aspirin dose had already been reduced, which argues against a purely salicylate mechanism. Both causes are self-limiting. The lesson for burn-associated KD is simply to monitor liver function throughout convalescence and promptly lower the aspirin dose if transaminases exceed five times normal.

### 3.1 Limitations

A single case cannot establish causation, and the short latency is compatible with coincidence. Coronary assessment used two-dimensional transthoracic echocardiography; follow-up currently extends to approximately 1 month after onset, at which point the coronary arteries remained normal without aneurysms. The wound was not recultured at the moment fever began, so transient colonization cannot be excluded. The literature review is descriptive, and the numbers are too small for statistical or causal inference; four Japanese-language cases summarized by Ueda et al. (12) were excluded because primary sources were unretrievable, and the pooled denominator could rise from 14 to 18 if they become available. Finally, we did not compare the observed frequency with the background KD incidence in East Asian children under 5 years of age (∼200–300/100,000/year) (16), a comparison that would be needed before any triggering role could be argued.

## 4 Conclusion

KD can develop during a pediatric burn, and this case shows that it may appear just as the wound re-epithelializes, precisely when a new fever is most likely to be dismissed as intercurrent infection. The specific, transferable lessons are as follows: consider KD in any burned child with unexplained persistent fever; re-examine all mucocutaneous surfaces, including limbs hidden by dressings; and, once KD is diagnosed, give IVIG promptly and monitor the coronary arteries (23) and liver function through convalescence. Whether burn injury is a genuine trigger or an incidental antecedent remains unresolved and will require a larger, systematically collected series.

## Data Availability

The datasets presented in this article are not readily available because of ethical and privacy restrictions. Requests to access the datasets should be directed to the corresponding author.
